# Electroactive Hydroxyapatite/Carbon Nanofiber Scaffolds for Osteogenic Differentiation of Human Adipose-Derived Stem Cells

**DOI:** 10.3390/ijms24010530

**Published:** 2022-12-28

**Authors:** Baojun Sun, Yajie Sun, Shuwei Han, Ruitong Zhang, Xiujuan Wang, Chunxia Meng, Tuo Ji, Chunhui Sun, Na Ren, Shaohua Ge, Hong Liu, Yang Yu, Jingang Wang

**Affiliations:** 1 Shandong Collaborative Innovation Center of Technology and Equipements for Biological Diagnosis and Therapy, Institute for Advanced Interdisciplinary Research (iAIR), University of Jinan, Jinan 250022, China; 2 Department of Periodontology, School and Hospital of Stomatology, Cheeloo College of Medicine, Shandong University & Shandong Key Laboratory of Oral Tissue Regeneration & Shandong Engineering Laboratory for Dental Materials and Oral Tissue Regeneration, Jinan 250012, China; 3 State Key Laboratory of Crystal Materials, Shandong University, Jinan 250100, China

**Keywords:** carbon nanofiber, hydroxyapatite, Ca^2+^, human adipose-derived mesenchymal stem cells, osteogenic differentiation

## Abstract

Traditional bone defect treatments are limited by an insufficient supply of autologous bone, the immune rejection of allogeneic bone grafts, and high medical costs. To address this medical need, bone tissue engineering has emerged as a promising option. Among the existing tissue engineering materials, the use of electroactive scaffolds has become a common strategy in bone repair. However, single-function electroactive scaffolds are not sufficient for scientific research or clinical application. On the other hand, multifunctional electroactive scaffolds are often complicated and expensive to prepare. Therefore, we propose a new tissue engineering strategy that optimizes the electrical properties and biocompatibility of carbon-based materials. Here, a hydroxyapatite/carbon nanofiber (HAp/CNF) scaffold with optimal electrical activity was prepared by electrospinning HAp nanoparticle-incorporated polyvinylidene fluoride (PVDF) and then carbonizing the fibers. Biochemical assessments of the markers of osteogenesis in human adipose-derived stem cells (h-ADSCs) cultured on HAp/CNF scaffolds demonstrate that the material promoted the osteogenic differentiation of h-ADSCs in the absence of an osteogenic factor. The results of this study show that electroactive carbon materials with a fibrous structure can promote the osteogenic differentiation of h-ADSCs, providing a new strategy for the preparation and application of carbon-based materials in bone tissue engineering.

## 1. Introduction

Bone defects are gaps formed by erosion caused by severe trauma, infection, or other deleterious events. When the size of the bone defect exceeds the body’s ability to heal spontaneously, clinical treatment is needed [1]. At present, multiple problems negatively impact the effectiveness of transplantation treatment. These issues include immune rejection and a shortage of donors, which make finding appropriate tissue a challenge, especially considering the narrow time window [2,3]. 

Considering the limitations of transplantation, bone tissue engineering has been pursued as an effective alternative method to repair bone defects. Due to the bioelectric properties of native bone tissue, electroactive scaffolds loaded with different types of cells and growth factors have been used in the regeneration of bone defects [4,5]. Carbon-based materials are readily available and tend to be biocompatible; such materials have been used in clinical treatments, such as in artificial valves and coronary stents [6,7]. Multiple studies have demonstrated that the structure and surface charge of carbon-based materials, such as graphene and carbon nanotubes, can affect the fate of stem cells [8,9,10]. Most carbon materials are deposited in the form of powders or nanolayers on other materials, such as glass, using the methods of vapor deposition and laser ablation, which limits their application [11,12,13]. 

Carbon nanofibers have structures and mechanical properties that are similar to those of the natural extracellular matrix. At the same time, they also have excellent electrical activity. Carbon-based materials have been found to influence the adhesion and proliferation of cells [14], and they have thus been suggested to be potentially useful clinically in tissue repair applications. For example, Ryu et al. [15] demonstrated that carbonized polyacrylonitrile can induce the osteogenic differentiation of mesenchymal stem cells (MSCs) without the need for exogenous inducing factors in a mouse model of calvarial defects. The main factor leading to the enhancing of osteogenesis is that carbonized polyacrylonitrile adsorbs more fibronectin than carbon nanotubes do. The absorbing of fibronectin stimulates mechanotransduction signals through a pathway involving focal adhesion kinase and extracellular signal-regulated kinases. 

Carbon fiber scaffolds and electroactive scaffolds have been demonstrated to promote osteogenic differentiation. For instance, Naskar et al. [16] synthesized a silk fibroin/carbon nanofiber scaffold that has a unique electrical conductivity which allows it to adapt to the piezoelectric properties of bone to induce the osteogenic differentiation of MSCs cultured on a three-dimensional scaffold. Pournaqi et al. [17] investigated the effect of a polyaniline composite scaffold on stem cell differentiation; they determined that a polyaniline/polyethersulfone conductive fiber scaffold was more effective at promoting the osteogenic differentiation of MSCs than a polyethersulfone-based scaffold. The explanation for this difference is that the optimal electrical conductivity of polyaniline creates a surface charge that is amenable to the stimulation of cellular activities including cell proliferation. 

Whereas carbon-based scaffolds have been used successfully to enhance cellular differentiation, the doping of these materials with other chemicals may provide additional benefits. Hydroxyapatite (HAp) is the main component of natural bone tissue [18]. Studies show that HAp can regulate the osteogenic differentiation of stem cells by releasing Ca^2+^ [19,20]. The increase in extracellular Ca^2+^ concentrations leads to a dramatic increase in intracellular Ca^2+^ concentrations. In turn, the intracellular Ca^2+^ can activate signaling pathways, including those mediated by calcium/calmodulin-dependent protein kinase, which play key roles in multiple aspects of osteoblast proliferation and differentiation [21,22]. 

In developing materials for use in bone engineering, the source of the stem cells is an important consideration. Bone marrow mesenchymal stem cells (BMSCs) are the most important source of cells in bone tissue engineering; however, as people age, the number and activity of BMSCs decreases significantly, making these cells unsuitable for the collection and extraction of autologous stem cells. Human adipose-derived stem cells (h-ADSCs), on the other hand, have a wide range of sources; their concentration in the plasma is more than 500 times higher than that of BMSCs, and they are relatively easy to extract [23,24,25,26,27]. Therefore, the use of h-ADSCs as seed cells has a stronger clinical potential.

Based on the importance of both electroactivity and Ca^2+^ release in bone engineering, integrating electroactive carbon-based materials with Ca^2+^-releasing HAp nanostructures may provide a practical approach to inducing osteogenic differentiation of hADSCs without the need for biological or chemical induction. However, the preparation of such a multifunctional material has remained a challenge. In this study, we discuss a novel method to fabricate HAp-carbon hybrid nanofibers with an electroactivity that is compatible with use in biological applications, and this material was used as a cell scaffold to investigate the osteogenic differentiation of h-ADSCs in the absence of an osteogenic inducing factor. These nanofibers were prepared using an electrospinning method with an HAp-dispersed polyvinylidene fluoride (PVDF) solution as a precursor. The nanofibers were further processed using carbonization. In general, polyacrylonitrile (PAN) is the most popular polymer for making carbon (nano) fibers. However, the concentrated heat release in the pre-oxidation stage of PAN can easily lead to melting and shrinkage, so it is necessary to apply traction and other means to ensure the properties of the fiber [28]. This leads to the complexity of the process. In this study, using PVDF fiber as the precursor, a carbon fiber with good properties can be obtained using defluorination, cross-linking, and direct carbonization. Furthermore, PVDF has the characteristics of removing HF in alkaline solutions and under heating conditions. After the removal of C and H with 62.5% mass fraction in PVDF, carbon fibers with rich pore structure will be obtained, which is conducive to the dissolution of Ca^2+^ in HAp. Therefore, the hydroxyapatite/carbon nanofiber films (HAp/CNF) prepared using this method were found to have a controllable pore size distribution, high electroactivity, large specific surface area, and controllable release of Ca^2+^. 

## 2. Results and Discussion 

### 2.1. Characterization of CNF and HAp/CNF

**Figure 1:**
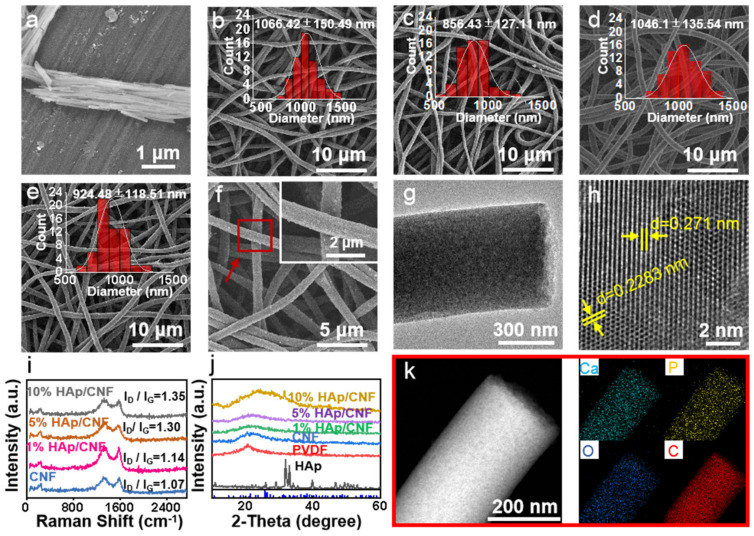
(**a**) SEM image of HAp. (**b**) SEM image of CNF. (**c**) SEM image of 1% HAp/CNF. (**d**) SEM image of 5% HAp/CNF. (**e**) SEM image of 10% HAp/CNF with the statistical distribution of fiber diameter fitted using Gaussian curve in the insets. (**f**) SEM images of 10% HAp/CNF at different magnifications. (**g**,**h**) TEM images of 5% HAp/CNF at different magnifications. (**i**) Raman spectrum of CNF and HAp/CNF with different concentrations of HAp. (**j**) XRD patterns of CNF and HAp/CNF with different concentrations of HAp. (**k**) EDS mapping images of HAp/CNF.

The morphologies of PVDF and HAp/CNF at the micro- and nano-scales were characterized using SEM. Although agglomeration occurred after drying, the HAp after treatment with ultrasound and by grinding was observed to have a nanorod-like structure with a diameter of 30 to 60 nm (Figure 1a). This structure was determined to be suitable for doping in solution for electrospinning. The PVDF fiber membrane prepared using electrospinning had a uniform fiber structure with an average diameter of 600 to 900 nm (Figure S1). Comparing the PVDF fibers with different HAp content, it can be found that the HAp content has no obvious effect on the fiber diameter. This is because the diameter of the PVDF fiber is mainly affected by spinning conditions, such as solution concentration, voltage, drawing distance, temperature, etc. HAp nanorods with a diameter of 30~60 nm will not significantly change the fiber diameter.

It is necessary to carry out the defluorination and cross-linking of the HAp/PVDF membrane before carbonization. As shown in Figure S2a, when the pure PVDF film was carbonized directly under the protection of argon, the PVDF film was deformed and broken without any mechanical strength. This is due to the fact that PVDF is first melted and then carbonized during the heating process. In order to prevent the hot melt of PVDF, it was cross-linked first. Our previous work [29] has shown that when PVDF was in an alkaline solution, part of F and H dissolved out in the form of HF, and a C=C bond was formed in the PVDF. In this work, a cross-linking agent was added at the same time of defluorination to make the C=C open and cross-link to each other, and a three-dimensional reticular structure was obtained. This structure has special stability, and the PVDF fiber membrane will not melt even after high temperature carbonization, and the carbon fiber membrane with a complete structure and certain mechanical strength is obtained (Figure S2b).

After carbonization, the prepared HAp/CNF exhibited a complete three-dimensional (3D) fiber network structure. Compared with the PVDF and HAp/PVDF film, the fibers of the CNF and HAp/CNF expanded slightly after carbonization (Figure 1b–e and Figure S1), which may be related to the removal of HF from the PVDF at high temperatures. Figure S3 shows the EDS images of PVDF before and after defluorination. It indicates that there is still a large amount of F in the samples after defluorination. In the carbonization process, the remaining F and H will overflow in the form of HF gas (as shown in Figure 1k, there was no F at all in the carbon fiber), resulting in the slight expansion of the fiber. Similar to HAp/PVDF, there was no significant difference in the fiber diameter with different HAp content after carbonization. At the same time, it was observed at high magnification that the fiber surfaces of CNF (Figure 1b) and HAp/CNF (Figure 1c–e) were rough compared to the surface of PVDF (Figure S1a). 

The crystal phase of HAp/CNF was analyzed using TEM (Figure 1g,h and Figure S4). The lattice spacings of HAp/CNF were measured to be 0.271 and 0.2283 nm (Figure 1h), which is consistent with the (310) plane (d = 0.262 nm) and the (221) plane (d = 0.2280) of Hap, as determined by the Joint Committee on Powder Diffraction Standards (JCPDS No. 09-0432). A lattice spacing of 0.248 nm was also observed in the HAp/CNF (Figure S4); this spacing is consistent with that of the (100) plane of graphite (d = 0.25 nm) [30]. Therefore, it can be concluded that the HAp/PVDF membrane underwent partial graphitization during the carbonization process, as indicated by the red squares in Figure S4. 

In addition, because of the removal of HF (the mass fraction was 62.5% in the PVDF) during carbonization, the carbonized fiber had a loose porous structure (Figure 1g), which was predicted to be beneficial to the dissolution of HAp in the fiber and the release of Ca^2+^. To detect the degree of graphitization of the carbon fibers, different materials were analyzed using Raman spectroscopy. Raman images of all materials showed two distinct D and G bands at 1320 and 1590 cm^−1^, respectively. With the increase in the amount of doped HAp, I_D_/I_G_ gradually increased from 1.07 to 1.35, indicating that HAp doping increased the exposure of defects and that this exposure led to the formation of amorphous carbon (Figure 1i). 

XRD was also performed to investigate the crystal structures of CNF as well as HAp/CNF containing 1, 5, and 10% HAp. As shown in Figure 1j, the main peak of the β phase of PVDF transformed into an amorphous carbon peak at 20.6° after carbonization. The XRD spectrum of the synthesized HAp is shown in Figure 1j. According to the standard card JCPDS No. 09-0432, the diffraction peaks at 25.8, 31.8, 32.9, 39.8, and 40.5° were identified as originating from the (002), (211), (300), (310), and (221) planes of HAp, respectively. This correlation confirmed that the HAp nanowires obtained using the hydrothermal method belonged to the hexagonal crystal system. Furthermore, a HAp diffraction peak at 32.9° can be seen in the HAp-doped material, especially in HAp/CNF with 10% HAp doping (Figure 1j). The energy dispersive spectroscopy (EDS) elemental mapping results of HAp/CNF (Figure 1k), indicate that Ca and P were uniformly distributed on HAp/CNF. Taken together, these results indicate that HAp had been successfully doped into CNF.

It has been confirmed that scaffolds with electrical activity can promote osteogenic differentiation [31,32]. Considering the good electrical conductivity of carbon-based materials, the electrochemical impedance spectroscopy (EIS) properties of the materials in PBS were measured with an electrochemical workstation. As shown in Figure S5, CNF and the three HAp/CNF materials showed electrochemical activity, and the impedance spectra were semicircular. The R_ct_ values of CNF and HAp/CNF with 1, 5, and 10% HAp were 10.4, 13.3, 9.2, and 12.8 Ω, respectively. The consistency of these values showed that doping with HAp had almost no effect on the R_ct_ value of the carbon fiber. Therefore, we concluded that CNF and HAp/CNF have excellent electrical activity and thus have the potential to regulate stem cell differentiation. 

As the hydrophilicity of materials can influence the adhesion and migration of cells [33], we evaluated the hydrophilic performance of the membranes using contact angle tests. As shown in Figure S6a, the contact angle of the CNF membranes was 29.28° and that of the HAp/CNF (5%) membrane was 26.09°. These results indicate that both CNF and HAp/CNF have high hydrophilicity, which would be beneficial for cell adhesion and migration. 

In short, these results demonstrate that CNF and HAp/CNF were successfully prepared, and the materials had excellent electroactivity. In addition, CNF and HAp/CNF with different HAp concentrations have similar degrees of graphitization and fiber structural properties. Therefore, the CNF and HAp/CNF prepared in this experiment were further evaluated for their ability to induce the osteogenic differentiation of h-ADSCs.

### 2.2. Viability and Proliferation of h-ADSCs on the Synthesized Membranes

To assess the cytotoxicity of the membranes, h-ADSCs were cultured on different samples, and cell proliferation was assessed using CCK-8 assays (S1), with cells cultured on a tissue culture plate serving as a control. The results shown in Figure S6b indicate that the viability of h-ADSCs cultured on the fiber membranes exhibited similar proliferation trends from day 1 to 5. On the first day, the viability of the cells on the membranes was only 50 to 70% of that of the control cells. On the third day, the viability of all cells was increased, and the viabilities of the cells cultured on the HAp/CNF membranes were approximately 60 to 80% of the control cells. This improvement indicated that the cells adapted to the fiber structures. The numbers of cells in all of the treatment groups increased steadily through the fifth day, and the total number of cells was approximately 2.8-fold higher on day 5 than on day 1. Specifically, the numbers of cells growing on the HAp/CNF membranes were approximately 85% of that of the control.

From the proliferation trends of cells, the materials have no obvious toxicity to cells. The slightly lower cell viability on the materials relative to the control may be attributed to the composition of the fiber membranes. As the membranes were composed of calcium and carbon, they may have promoted cell differentiation rather than cell proliferation. Previous studies have confirmed that decreasing cell proliferation is usually related to cell senescence, apoptosis, and differentiation [34,35]. Therefore, the lower cell proliferation on the materials may indicate enhanced osteogenic differentiation of the cells. 

**Figure 2:**
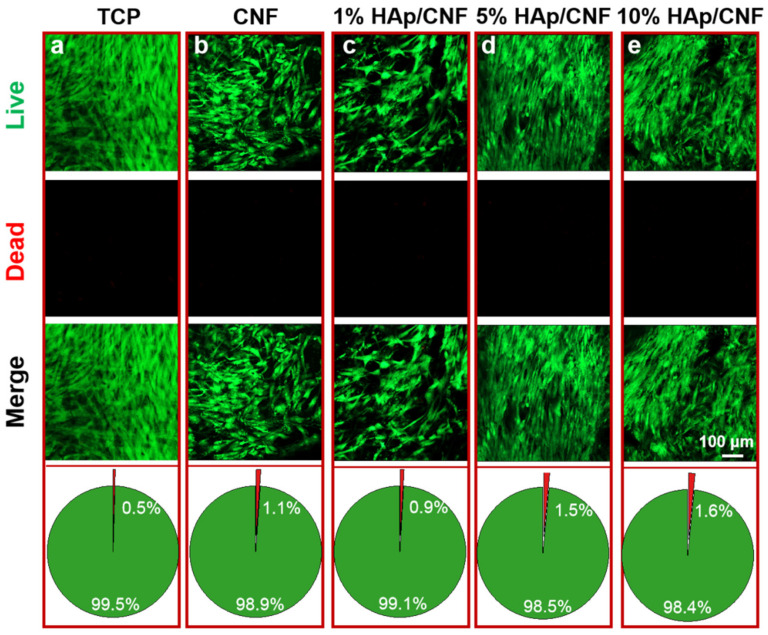
Live/dead cell staining and analysis of the proportion of live and dead cells cultured on various materials after 2 days. (**a**) TCP: tissue culture plates. (**b**) CNF membranes. (**c**) 1% HAp/CNF membranes. (**d**) 5% HAp/CNF membranes. (**e**) 10% HAp/CNF membranes. Live cells were stained green, and the dead cells were stained red.

To further investigate the cytocompatibility of the membranes, an assay that detects live and dead cells was used to perform a quantitative evaluation. In this series of experiments, cells grown on the three HAp/CNF membranes with different HAp concentrations were used as the experimental groups, and cells grown on tissue culture plates were used as a control group. As shown in Figure 2, after 2 days of culture, the live cells were stained green, and the dead cells were dyed red. The cells cultured under all conditions remained alive, and no dead cells were observed. The statistical analyses show that the percentage of dead cells in all trials was lower than 1.6%, suggesting that the HAp/CNF membranes were biocompatible with h-ADSCs and were not toxic to these cells. Therefore, we concluded that these materials were suitable for cell culture.

### 2.3. Morphologies of Adherent and Spreading h-ADSCs

**Figure 3:**
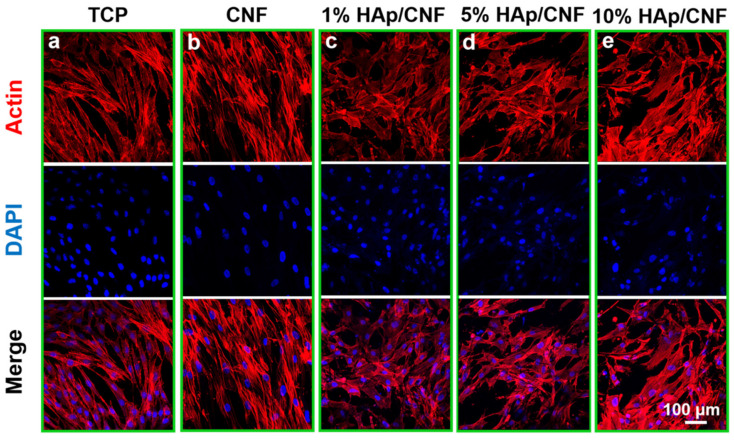
Adhesion and spreading of h-ADSCs on the surface of different materials after 7 days: (**a**) TCP. (**b**) CNF. (**c**) 1% HAp/CNF. (**d**) 5% HAp/CNF. (**e**) 10% HAp/CNF. Red indicates F-actin, as stained with rhodamine phalloidin, and nuclei were stained blue with DAPI. TCP: tissue culture plastic, CNF: carbonized fibers, HAp: hydroxyapatite.

Immunofluorescence staining of F-actin was used to observe the cell morphologies of adhesive cells with different materials as substrates (Figure 3). After 7 days of culture, the adhesion morphologies of the control h-ADSCs maintained the typical spindle shape of stem cells. However, the cells cultured on CNF (Figure 3b) and HAp/CNF (Figure 3c–e) presented polygonal morphologies with widening cell skeletons and pseudopodia, which are consistent with osteogenic differentiation. Moreover, in cells cultured on CNF and HAp/CNF, pseudopodia that spread along the nanofibers were observed; the pseudopodia produced by h-ADSCs cultured on the HAp/CNF materials were more prominent. 

To further investigate these changes, SEM was used to analyze the morphological changes of h-ADSCs after culturing on different materials for 7 days. As shown in Figure S7, the control h-ADSCs maintained a long spindle shape with elongated cell pseudopodia. On the other hand, cells cultured on the CNF and HAp/CNF membranes had distinct osteoblast-like polygonal shapes. The pseudopodia of cells cultured on HAp/CNF stretched along the nanofibers, and the cells were polygonal. These results were consistent with the results of F-actin immunofluorescence staining in that the cells cultured on the HAp/CNF membranes appeared to adhere and spread more readily than those cultured on other materials. 

These morphological analyses reveal that the CNF and HAp/CNF membranes were biocompatible with h-ADSCs. In addition, these membranes, especially those containing HAp, were found to have the ability to influence the morphologies of h-ADSCs. Therefore, we speculated that the rough carbon fiber structures with certain electrical properties would promote the osteogenic differentiation of h-ADSCs. With the addition of HAp, the synergistic effect of Ca^2+^ would accelerate the process of osteogenic differentiation.

### 2.4. Osteogenic Differentiation of h-ADSCs

#### 2.4.1. Alkaline Phosphatase Activity

**Figure 4:**
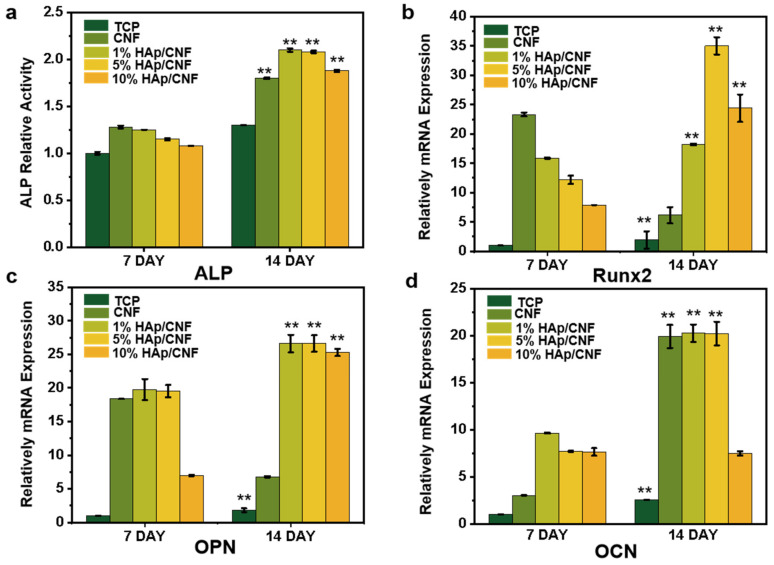
Biochemical markers of osteogenic differentiation of h-ADSCs cultured on CNF and HAp/CNF membranes. (**a**) Alkaline phosphatase (ALP) activity in extracts of h-ADSCs on days 7 and 14 of culturing on the noted materials. (**b**) Runt-related transcription factor 2 (*RUNX2*), (**c**) osteopontin (*OPN*), and (**d**) osteocalcin (*OCN*). All data are shown as means ± standard deviation (n = 3). ** *p* < 0.01.

Alkaline phosphatase (ALP) is an early phenotypic marker of osteogenic differentiation and can be used to assess the degree of the osteogenic differentiation of stem cells [36,37]. To evaluate whether the h-ADSCs cultured on different materials had undergone osteogenic differentiation, an ALP assay was conducted on extracts from cells cultured appropriately (S2.1). Figure 4a shows the relative activity of ALP normalized to the total protein content of h-ADSCs cultured on different materials. Although the ALP activity of cells grown on the HAp/CNF membranes was not clearly different from that of cells cultured on a CNF membrane after 7 days, the activity of ALP in the cells grown on HAp/CNF and CNF membranes materials was slightly higher than that of the control cells. However, after 14 d of culturing, the ALP activity of cells cultured under control conditions increased approximately 1.3-fold compared with that of the control cells cultured for 7 d. Meanwhile, the ALP activity of the cells cultured for 14 d on CNF was 1.4-fold higher than that of the cells cultured under control conditions for the same amount of time. The ALP activity of cells grown on 1, 5, and 10% HAp/CNF were approximately 1.6-, 1.6-, and 1.45-fold higher, respectively, than that of the control cells cultured for 14 d. The larger increases in ALP in the cells cultured on CNF and HAp/CNF are consistent with the enhancing effect of these materials on osteogenic differentiation. In addition, the tendency of the materials to promote the osteogenic differentiation of cells was more obvious after doping with HAp. Notably, the fiber membranes doped with a relatively small amount of HAp (1 or 5%) led to larger increases in ALP expression than the material doped with 10% HAp.

#### 2.4.2. Expression of Genes Related to Osteogenesis

The trend in the osteogenic differentiation of h-ADSCs cultured on different materials was further investigated by analyzing the expression of osteogenesis-related genes using q-PCR (S2.2). *RUNX2* encodes runt-related transcription factor 2, which regulates osteoblast differentiation and plays an important role in bone formation, cartilage formation, and bone metabolism [38,39]. Osteopontin, which is encoded by *OPN*, is a glycoprotein that plays a key role in bone matrix mineralization [40,41]. Osteocalcin, encoded by *OCN*, is important in the process of osteogenic differentiation and is considered to be a marker of the mineralization stage, which generally occurs at the end of osteoblast differentiation [42]. 

As shown in Figure 4b, the expression of *RUNX2* in h-ADSCs cultured on the CNF and different HAp/CNF materials for 7 days was substantially enhanced compared with the expression in the control cells. The culturing of cells on CNF, and 1, 5, and 10% HAp/CNF led to increases in *RUNX2* expression of 23.3-, 15.8-, 12.2-, and 7.9-fold, respectively, relative to the expression in cells grown on tissue culture plastic. 

After 14 days of culture, the expression of the *RUNX2* gene in cells grown on CNF, and 1, 5, and 10% HAp/CNF was increased 6.1-, 18.2-, 35-, and 24.4-fold, respectively, relative to the expression in cells grown under control conditions. The decrease in *RUNX2* expression in cells grown on CNF correlated with an increase in the relative expression of *OCN* and was thus likely related to the entering of the final stage of osteogenic differentiation. Due to the lack of a source of Ca^2+^, the expression of *RUNX2* could not be efficiently promoted after 14 days, so the expression of *RUNX2* decreased. 

As shown in Figure 4c, the trends in the expression of *OPN* in cells cultured on CNF and HAp/CNF materials were similar to those of *OCN*. In both cases, the expression levels of these genes were clearly increased in cells grown on synthetic materials compared to those grown on tissue culture plastic. At day 7 of culturing, the expression of *OPN* in cells cultured on CNF, and 1, 5, and 10% HAp/CNF were higher by 18.3-, 19.7-, 19.5-, and 7.0-fold, respectively, compared with expression in the control cells. In addition, the expression of *OPN* in cells cultured on different fiber membranes continued to increase through to day 14. The levels of expression of *OPN* in the cells grown on those materials were 6.8-, 26.6-, 26.6-, and 25.3-fold higher, respectively, than those of the control cells on day 7. The expression of *OCN* in cells grown on CNF was 2.5-fold higher than that of the control cells on day 7, whereas the gene expression levels in 1, 5, and 10% HAp/CNF were 9.6-, 7.7-, and 7.6-fold, relative to the control. The levels of expression of *OCN* in cells grown on CNF, and 1, 5, and 10% HAp/CNF were higher than those of the control cells after 14 days of culture. Compared with the control cells on day 7, the expression levels of both genes in the cells grown on CNF, and 1, 5, and 10% HAp/CNF on day 14 were increased by 19-, 20.3-, 20.2-, and 7.5-fold, respectively. 

This investigation into the influence of the growth substrate on the expression of genes typical of osteogenic differentiation suggested that CNF and HAp/CNF can induce the osteogenic differentiation of h-ADSCs, whereas the h-ADSCs grown on tissue culture plastic remained almost undifferentiated. Compared with CNF, HAp/CNF promoted osteogenic differentiation more strongly, but the differentiation effect did not completely increase with the increase in HAp content. When the content of HAp increased to 10%, the differentiation ability of cells decreased to a certain extent. This may be related to the release of Ca^2+^. Higher concentrations of HAp will release more Ca^2+^ (Table S2), thus promoting the osteogenic differentiation of h-ADSCs, but too high a concentration of Ca^2+^ may lead to nuclear imbalance in cells [43], which may affect cell activity and differentiation. In this experiment, a concentration of HAp of 5% led to the strongest osteogenic differentiation. 

#### 2.4.3. Expression of Proteins Related to Osteogenesis

**Figure 5:**
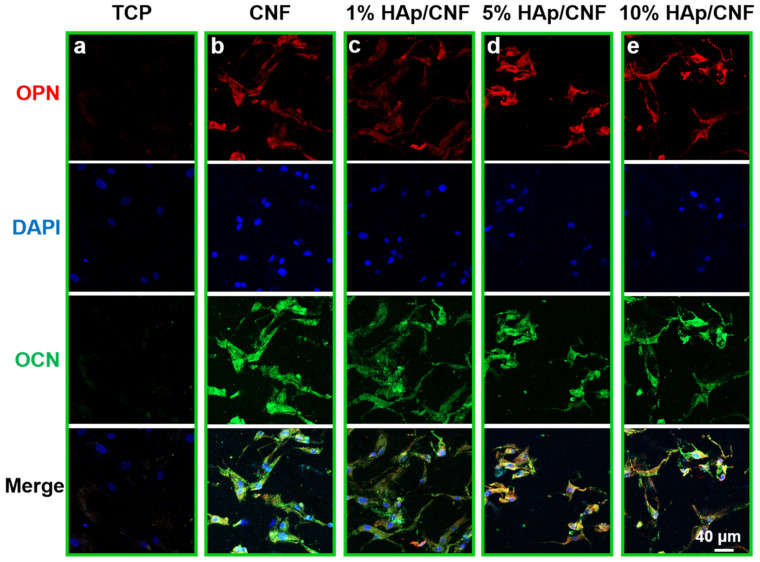
Immunofluorescent detection of proteins indicative of osteogenic differentiation in h-ADSCs cultured on different materials. Cells grown on (**a**) tissue culture plastic (TCP), (**b**) CNF, (**c**) 1% HAp/CNF, (**d**) 5% HAp/CNF, or (**e**) 10% HAp/CNF were fixed and stained with antibodies to the noted proteins. Red represents osteopontin (OPN), green represents osteocalcin (OCN), and blue represents nuclei.

To further investigate the level of osteogenic differentiation of h-ADSCs cultured on different materials, immunofluorescence staining was conducted to evaluate the expression of the osteogenic-specific proteins OCN and OPN at the protein level. As shown in Figure 5, after 21 days of culture, the results of the immunofluorescence staining assays were consistent with the results of the q-PCR assays. For example, the h-ADSCs cultured on tissue culture plastic exhibited weaker fluorescence intensities indicating OCN and OPN than cells grown on the fiber membranes. The intensity of the OCN- and OPN-related fluorescence of cells cultured on the fiber membranes presented were strong, indicating that OPN and OCN proteins were expressed at high levels in cells grown on the fiber membranes. More specifically, increasing HAp apparently led to the increased expression of OCN and OPN proteins relative to growth on CNF, with fiber membranes doped with 5% HAp leading to the strongest immunofluorescence intensities. 

Quantitative analyses of the immunofluorescence intensities were also performed (Figure S8). These analyses similarly demonstrate that growth on CNF and HAp/CNF led to higher fluorescence intensity compared with the control (Figure S8). Taken together, these results confirm that 5% HAp/CNF accelerated the osteogenic differentiation of h-ADSCs more than other fiber membranes. The expression of these genes at the mRNA and protein levels in cells grown on material containing 10% HAp was slightly lower than that of cells grown on the other HAp-containing materials on day 14. As noted above, this difference may be due to the fact that higher concentrations of Ca^2+^ affect the nuclear balance in the cell [44].

#### 2.4.4. Formation of Osteoblast Mineralized Calcium Nodules

Mineralized nodules are a marker of osteoblast differentiation and maturation, and the osteogenic function of osteoblasts. Accordingly, the observation of osteoblast mineralized nodules is a commonly used technique to verify osteoblast differentiation [44,45]. Here, cell surface Ca^2+^ deposits were visualized with Alizarin Red S (ARS) staining. As 5% HAp/CNF led to the strongest induction of osteogenic differentiation according to the assays of the expression of osteogenesis-related genes, 5% HAp/CNF was selected for this set of experiments. 

**Figure 6:**
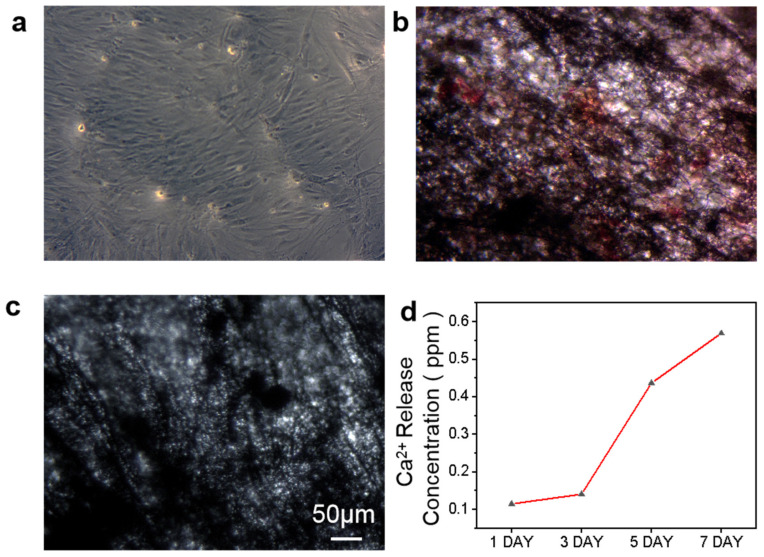
Alizarin Red S staining on day 21 shows osteoblast mineralized calcium nodules in cells grown on (**a**) tissue culture plastic (TCP) and (**b**) 5% HAp/CNF. Panel (**c**) shows staining of 5% HAp/CNF without cells. (**d**) The concentration of Ca^2+^ in solution in 7 days.

After culturing cells on tissue culture plastic and 5% HAp/CNF for 21 days, the extent of calcium deposition was assessed. As shown in Figure 6a,b, no mineralized nodules were evident on the control cells, whereas red calcium nodules were observed on the cells grown on the 5% HAp/CNF material. These results demonstrate that the hADSCs did not differentiate into osteoblasts spontaneously but instead needed to be induced to differentiate. In addition, we also stained 5% HAp/CNF membranes in the absence of cells to determine whether Ca^2+^ on the material could lead to false positive results in the staining assay, but no red-stained calcium nodules appeared (Figure 6c). These results further support the notion that 5% HAp/CNF promotes the Ca^2+^ secretion and maturation of h-ADSCs. 

### 2.5. The Effects of HAp/CNF on the Morphology of h-ADSCs In Vitro

As cultured cells directly contact the material of the substrate, the nanostructure of the material affects cell adhesion and migration, thus regulating cell differentiation. Different from flat surfaces, on a nanostructured matrix, stem cells can differentiate into functional cells with appropriate morphological structures according to the topological structure of the matrix [46,47]. Yin et al. [48] found that directional fibers can promote the differentiation of MSCs into tendon tissue, whereas random fibers make the cytoskeleton polygonal and promote the osteogenic differentiation of MSCs. 

The surface roughness of materials is also an important factor affecting stem cell differentiation. Chen et al. [49] showed that a rough surface is beneficial to the osteogenic differentiation of cells, whereas a smooth surface maintains the stemness of the cells. Here, the SEM images (Figure 1b–e) demonstrate that CNF and HAp/CNF nanofibers have three-dimensional structures and rough surfaces. When stem cells migrate on these materials, the fibers likely undergo mechanical changes, affecting cell morphology and adhesion, thus promoting the osteogenic differentiation of cells. 

In addition, the pseudopodia of cells on HAp/CNF (Figure 3c–e) surfaces are broader than those of cells on CNF (Figure 3b). The different broadness of these structures may be due to the release of Ca^2+^ from the composite carbon fibers. Previous studies have shown that Ca^2+^ can activate proteolytic enzymes through L-type calcium channels [50,51], which mediated the expression of OCN and OPN, thus accelerating the osteogenic differentiation of h-ADSCs. The concentration of Ca^2+^ released by HAp/CNF in the culture medium over time was determined using ICP-MS (Table S2). This assay demonstrated that the concentration of Ca^2+^ in the culture medium increased upon incubation with this material; the Ca^2+^ concentration on day 7 was approximately 5-fold higher than when the material was initially added to the medium. Ca^2+^ may be released upon the decomposition of the HAp doped in the fiber in solution, and the release results in a gradual increase in the concentration of Ca^2+^ in the microenvironment of the cells.

In addition, as demonstrated by Jing et al. [52], the surface charge of conductive fibers plays an important role in regulating protein adsorption, ion migration, and nucleation. These factors are especially strong when the surface charge of HAp/CNF is created, and the intracellular calcium increases, thus accelerating the osteogenic differentiation of seed cells. Therefore, a fiber scaffold with electrical activity, such as that described in this paper, would facilitate the opening of voltage-gated calcium channels [53]. The resulting influx of Ca^2+^ released from the fiber would support mineralization of the cell matrix and promote differentiation into osteoblasts. Comparing the bone differentiation of stem cells on carbon fiber surface with and without HAp addition, we propose that Ca^2+^ and electroactive carbon fibers synergistically promote the development and maturation of osteoblasts.

## 3. Materials and Methods 

### 3.1. Materials

The following chemicals were used to synthesize CNF and HAp/CNF. PVDF powder (Solvay 6010) was purchased from commercial sources. Oleic acid, calcium chloride (CaCl_2_), sodium hydroxide (NaOH), sodium dihydrogen phosphate dihydrate (NaH_2_PO_4_·2H_2_O), 1, 4-Bis (aminomethyl) benzene, absolute ethanol, methanol, nitric acid, N-hexane, N,N-dimethylacetamide (DMF), and acetone were purchased from Sinopharm (Shanghai, China) and can be used directly without any further purification. Deionized water was used throughout this study.

For cell experiments, α-minimum essential medium (α-MEM) and fetal bovine serum (FBS) were provided by Gibco (Thermo Fisher Scientific, Waltham, MA, USA). Penicillin-streptomycin (penicillin 10,000 IU/mL and streptomycin 10 mg/mL) and phosphate buffered saline (PBS) (1×) pH 7.2 were purchased from SparkJade (Qingdao, China). In addition, 4% paraformaldehyde (Beyotime, Shanghai, China), 2.5% glutaraldehyde (Macklin, Shanghai, China), Triton X-100, bovine serum albumin (BSA), BCA protein assay kit, ALP activity assay kit, and 2% Alizarin Red S (ARS) were purchased from Solarbio (Beijing, China). Cell Counting Kit-8 (CCK-8) and rhodamine phalloidin were purchased from MedChemExpress (Shanghai, China) and Cytoskeleton (Denver, USA), respectively. Primary antibodies for osteocalcin (OCN) and osteopontin (OPN) were purchased from Proteintech (HuBei, China). Trizol reagent, 4′, 6-diamidino-2-phenylindole (DAPI), live/dead viability/cytotoxicity kit, and OCN and OPN secondary antibodies were purchased from Invitrogen (Waltham, MA, USA).

### 3.2. Electrospinning of HAp/PVDF Composite Membranes

PVDF membranes were prepared as previously described [54]. Briefly, PVDF powder was dissolved in a mixed solution composed of N,N-dimethylacetamide and acetone (2:3, vol:vol), so that the final concentration was 12 wt.%. The mixture was stirred until it became a transparent solution. Spinning solutions were prepared to produce different mass ratios of HAp to PVDF. These solutions included PVDF without HAp, and 1, 5, and 10% HAp/PVDF. These materials are collectively referred to as HAp/PVDF unless otherwise specified. Then, the mixed solution was pulled into a 5 mL syringe through an opening with an inner diameter of 0.5 mm. A voltage of 20 kV was applied across a collection distance of 15 cm, and the electrospun fibers were extruded at a rate of 0.6 mL/h. The preparation of the mixtures and the process of electrospinning were carried out at room temperature. The electrospinning process was completed with a PEG-1 electrospinning machine (Qingdao Junda Technology Co., Ltd., Qingdao, China). Finally, the prepared fiber membranes were dried in an oven at 60 °C for 30 min. 

### 3.3. Preparation of CNF and HAp/CNF Membranes

The membranes were subjected to cross-linking prior to high-temperature carbonization [55]. Next, 1,4-Bis(aminomethyl)benzene (4 g) and MgO nanoparticles (2 g) were dispersed into methanol (15 g), and then NaOH (1 g) was added to the mixture. A beaker containing a fiber membrane in this mixture was placed on an oscillator to shake at room temperature for 44 h, when the cross-linking reaction was presumed to be completed. Then, the membranes were soaked in 1 M HNO_3_ for 1 h, followed by a 6 h incubation in deionized water, a 2 h incubation in anhydrous methanol, and a 2 h incubation in N-hexane. This series of washes was repeated three times to remove residual byproduct particles.

After cross-linking, the PVDF and HAp/PVDF nanofiber membranes were placed in a tubular furnace filled with argon. The furnace temperature was increased to 1000 °C at a rate of increase of 5 °C/min to produce the CNF and HAp/CNF membranes.

### 3.4. Characterization

Scanning electron microscopy (SEM), performed using a Regulus 8100 system (Hitachi High-Technologies Corporation), was used to characterize the material morphologies. Transmission electron microscopy (TEM), performed using an FEI Talos F200X system, was used to characterize lattices and perform elemental analyses of the materials. X-ray diffraction (XRD) information was recorded using a Rigaku Ultima IV X-ray diffractometer with CuKα (λ = 0.15406 nm) irradiation. The water contact angle of the samples was measured using a JC2000D2G contact angle measuring instrument (Shanghai Zhongchen Digital Technology Equipment Co., Ltd., Shanghai, China). The degree of graphitization of CNF was examined using Raman spectroscopy (LabRAM HR Evolution, Horiba, France). The resistivity was measured with an electrochemical workstation (CHI760E, Brilliance, China). 

### 3.5. Cell Culture 

Cryopreserved h-ADSCs (provided by the Department of Orthopedics, Qilu Hospital of Shandong Province) were rapidly thawed, recovered in a 37 °C water bath, and cultured in 10 mL of α-minimal essential medium containing 1% penicillin–streptomycin and 10% FBS. After culturing the cells for 24 h at 37 °C in a humidified atmosphere with 5% CO_2_, the medium was replaced with fresh complete growth medium, and the medium was then changed every two days. Details regarding the evaluation of cytocompatibility of CNF and HAp/CNF and the evaluation of osteogenic differentiation are available in the Supplementary Materials.

### 3.6. Ca^2+^ Release Measurements

HAp/CNF membranes of the same size were placed in centrifuge tubes containing 3 mL of culture medium. The culture medium from days 1, 3, 5, and 7 were used to measure the Ca^2+^ concentration using inductively coupled plasma mass spectrometry (ICP-MS) (Agilent ICPMS 7700).

## 4. Conclusions

HAp-doped PVDF fibers were prepared by electrospinning, and the fibrous bone tissue engineering scaffold material (HAp/CNF) was obtained after carbonization. The material has high electrical conductivity and a porous structure, and Ca^2+^ is released gradually from the HAp-doped fiber along the porous structure. The results of the measurements of the differentiation of h-ADSCs show that these cells did not differentiate spontaneously on the tissue culture plastic, but they differentiated into osteocytes spontaneously on carbon nanowires. The induction of the osteogenic differentiation of h-ADSCs is due to the electrical activity of the material. Compared with CNF, HAp/CNF has a higher ability to induce the osteogenic differentiation of h-ADSCs. This ability comes from the combined action of the electrical activity of carbon fiber materials and the induction of Ca^2+^ osteogenesis. Therefore, this paper describes an inexpensive, simple, and rapid method for the preparation of carbon fiber materials, and it provides a new method for the application of carbon fiber materials in bone tissue engineering.

## Data Availability

The data involved in this paper have been presented in articles and Supporting Materials in the form of diagrams or tables.

## Supplementary Materials

The following supporting information can be downloaded at: https://www.mdpi.com/article/10.3390/ijms24010530/s1.
