# Supplementary material for: Electroactive Hydroxyapatite/Carbon Nanofiber Scaffolds for Osteogenic Differentiation of Human Adipose-Derived Stem Cells
Source: Int J Mol Sci. 2022 Dec 28;24(1):530. doi: 10.3390/ijms24010530 (PMC9820130; doi:10.3390/ijms24010530)
Supplement: Supplementary file 1 [file ijms-24-00530-s001.zip › ijms-2061172-supplementary.pdf]

Electroactive Hydroxyapatite/Carbon Nanofiber Scaffolds for Osteogenic Differentiation of Human Adipose-Derived Stem Cells

Baojun Sun [1,†], Yajie Sun [1,†], Shuwei Han [1], Ruitong Zhang [1], Xiujuan Wang [1], Chunxia Meng [1], Tuo Ji [1], Chunhui Sun [1], Na Ren [1], Shaohua Ge [2], Hong Liu [1,3], Yang Yu [2,*] and Jingang Wang [1,*]

[a] Shandong Collaborative Innovation Center of Technology and Equipements for Biological Diagnosis and Therapy, Institute for Advanced Interdisciplinary Research

(IAIR), University of Jinan, Jinan 250022, PR China

[b] Department of Periodontology, School and Hospital of Stomatology, Cheeloo College of Medicine, Shandong University & Shandong Key Laboratory of Oral Tissue Regeneration & Shandong Engineering Laboratory for Dental Materials and Oral Tissue Regeneration, Jinan, Shandong, 250012, China

[c] State Key Laboratory of Crystal Materials, Shandong University, Jinan 250100, PR China

S1. Cytocompatibility Evaluation of CNF and HAp/CNF

The sample was cut into the same size to fit a 24-well plate. All the prepared samples were soaked in 75% ethanol and sterilized overnight with ultraviolet light. Then, 200 μL of cell suspension consisting of 2×10^4 cells were cultured on each sample in the 24-well plate, and 800 μL of complete medium

was added to each well. To observe the survival of the cells cultured on the surface of different samples, Live/Dead Assays of cells cultured for two days was performed. The cells cultured on the material and tissue culture plastic were incubated with 200 μL of basal medium containing 4 μM propidium iodide (PI) and 2 μM calcein AM at 37°C for 15 min. After washing with PBS, observation was performed under a laser microscope (LSM800, Zeiss, Germany).

To quantitatively estimate the cell viability, the CCK-8 assay was carried out according to the kit instructions. Cells were incubated with 10% CCK-8 in the basal medium for 2 h at 37°C. The level of water-soluble formazan dye was detected by using a microplate reader at a wavelength of 450 nm (SYNERGY H1; BioTek, USA). The above samples were all done in three replicates.

To observe the adhesion and morphology of the cells cultured on the samples, after 7 days of cultured, the cells were washed 3 times with PBS and fixed with 4% paraformaldehyde solution for 10 min. Then, the cells were permeabilized with 0.1% Triton X-100 for 5 minutes and blocked with 10% BSA for 30 minutes. The cell was stained with rhodamine phalloidin (1:200 dilution) for 30 minutes and incubated with DAPI (1:1000 dilution) to stain the nuclei for 10 minutes. Finally, the cells were washed 3 times with PBS and observed under the confocal laser microscope.

To further observe the attachment of h-ADSCs on CNF and HAp/CNF

membranes, after 7 days of cultured, h-ADSCs were washed 3 times with PBS and then soaked with 2.5% glutaraldehyde overnight. Cells were then graded dehydrated using a series of alcohol solutions (30%, 50%, 70%, 80%, 90%, 95%, 98%, and 100%) and frozen at -80°C for 6 h before using freeze dryer overnight. Finally, after spraying gold for 30 seconds under a current of 20 μA, the cells were observed by SEM.

S2. Osteogenic Differentiation of Evaluation of CNF and HAp/CNF

S2.1 Alkaline Phosphatase (ALP) Activity

After 7 and 14 days of cultured, h-ADSCs on different samples were lysed by cell lysis buffer to obtain total intracellular protein content and perform ALP activity assays. Total protein content was determined using the BCA protein assay by measuring the absorbance of the reaction solution at 570 nm. ALP activity was analyzed by using an ALP activity assay kit according to the manufacturer's instructions. Briefly, under alkaline conditions, ALP catalyzed the formation of free phenols by disodium phenyl phosphate. Phenol reacted with 4-Aminoantipyrine and potassium ferricyanide as a red subquinone derivative by optical measurement at a wavelength of 510 nm. Relative ALP normalized activity to the protein content of cultured cells on different samples (n = 3 per group).

S2.2 Quantitative Polymerase Chain Reaction (q-PCR)

After 1 day of culture, the complete medium containing 10% FBS was replaced with a medium containing 5% FBS without any osteogenic inducing factors. After 7, 14, and 21 days of culture, the samples were treated with TRIZOL reagent to extract total RNA on an ice plate. A real-time PCR system (LightCycler 96 Roche) was used for q-PCR analysis of OCN, OPN, and Runx2 gene expression (primer sequence in Table S1, support information). The target gene expression was normalized to the expression of β-actin and expressed as the mean ± SD (n = 3 per group). The above primers were purchased from Jinan Boshang Biotechnology Co., Ltd.

S2.3 Immunofluorescence Staining

After 21 days of culture, the cells were fixed with 4% paraformaldehyde for 10 minutes. Then, the cell membrane was permeabilized by using 0.1% Triton X-100 for 10 minutes, and the cells were blocked with a 10% BSA solution for 1 hour at room temperature. After blocking, the cells were incubated with primary antibodies OCN (rabbit polyclonal anti-OCN, Proteintech) and OPN (mouse monoclonal anti-OPN, Proteintech) with 1% BSA solution diluted 1:1000 at 4℃ overnight. Goat anti-rabbit Alexa Fluor 488 (diluted 1:1000) and goat anti-mouse Alexa Fluor 594 (diluted 1:1000) secondary antibodies in 1% BSA were stained for OCN and OPN for 1 hour at room temperature. PBS with Tween 20 (Sinopharm) was used to rinse off the secondary antibody. Then, the cell nucleus was stained with DAPI for 10

minutes and washed with PBS 3 times. The images of the stained sample were acquired under a laser confocal microscope.

S2.4 NAlizarin Red S Staining (ARS)

After 21 days of culture, h-ADSCs on CNF, 5% HAp/CNF, and tissue culture plastic were fixed in 4% paraformaldehyde for 30 min and then stained with 2% Alizarin Red S (pH=4.2) for 10 min. After rinsing three times with distilled water, the stained samples were observed with an inverted microscope (PrimoVert, Zeiss, Germany).

Table S1. q-PCR primer sequences.

Gene	Forward primers (5′-3′)	Reverse primers (5′-3′)
β-actin	CATGTACGTTGCTATCCAGGC	CTCCTTAATGTCACGCACGAT
OCN	AAGCCCAGCGACTCTGAGTCT	CCGGAGTCTATTCACCACCTTACT
OPN	TCCTGTCTCCCGGTGAAAGT	GGCTACAGCATCTGAGTGTTTGC
Runx2	AATGCCTCCGCTGTTATG	TTCTGTCTGTGCCTTCTTG

Table S2. The concentration of Ca^{2+} in the culture medium at different time.

Day	Element	Element Concentration C_0（mg/L)	Dilution Factor f	Digestion Solution/Original Sample Solution Element Concentration C_1（mg/L)
1	Ca	0.1145	1	0.1145
3	Ca	0.1401	1	0.1401
5	Ca	0.4361	1	0.4361
7	Ca	0.5686	1	0.5686

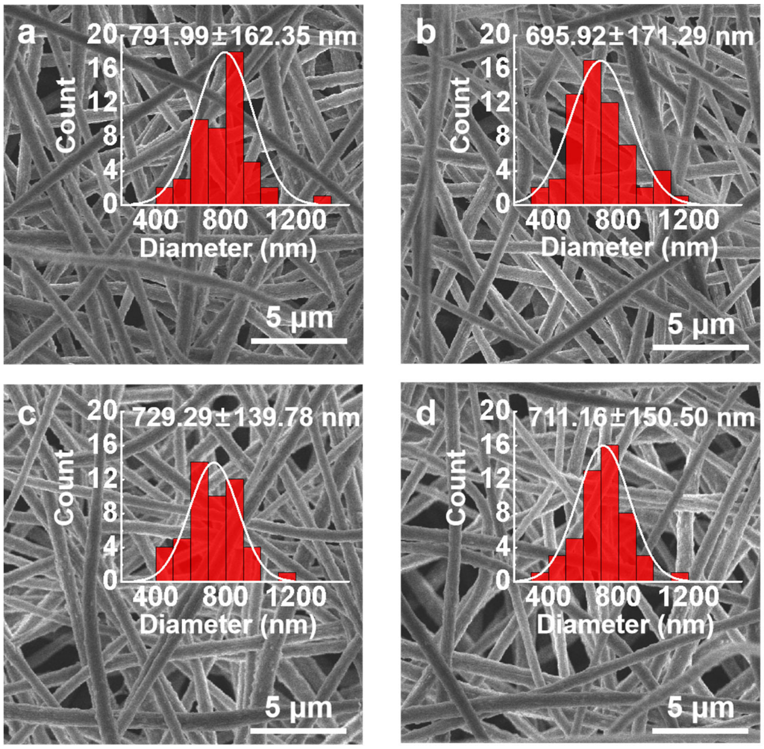

Figure S1. Characteristics of PVDF and HAp/PVDF. (**a**) SEM image of PVDF. (**b**) SEM image of 1% HAp/PVDF. (**c**) SEM image of 5% HAp/PVDF. (**d**) SEM image of 10% HAp/PVDF with their statistical distribution of fiber diameter fitted by Gaussian curve in the insets.

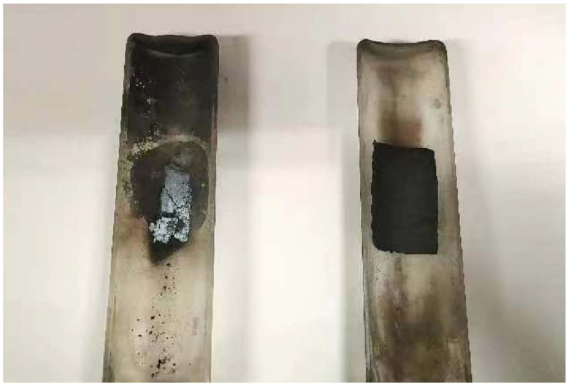

Figure S2. Comparison diagram of direct carbonization and defluorinated cross-linked carbonization.

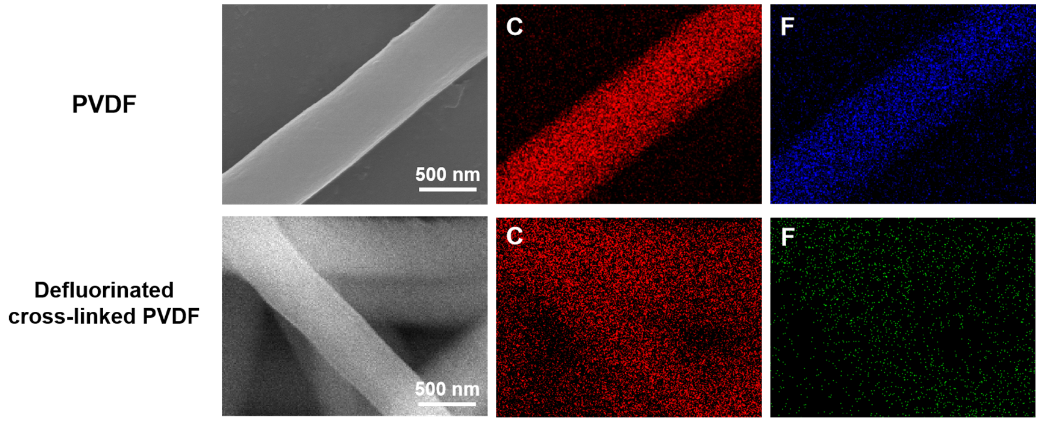

Figure S3. EDS images of PVDF before and after defluorinated cross-linked.

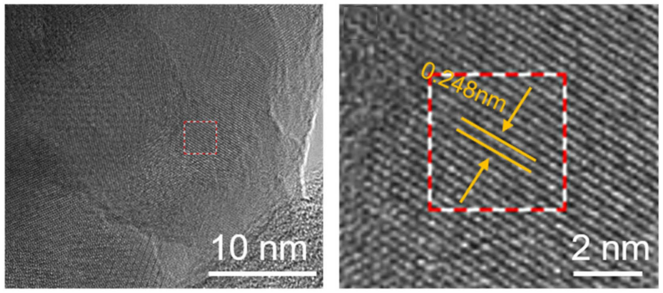

Figure S4. TEM image of HAp/CNF at different magnifications.

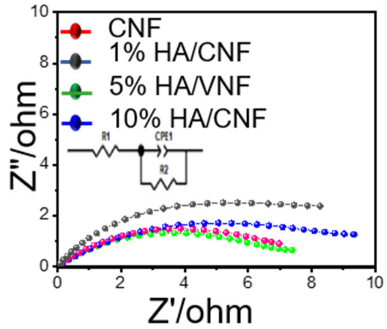

Figure S5. Electrical Impedance Spectra of different materials.

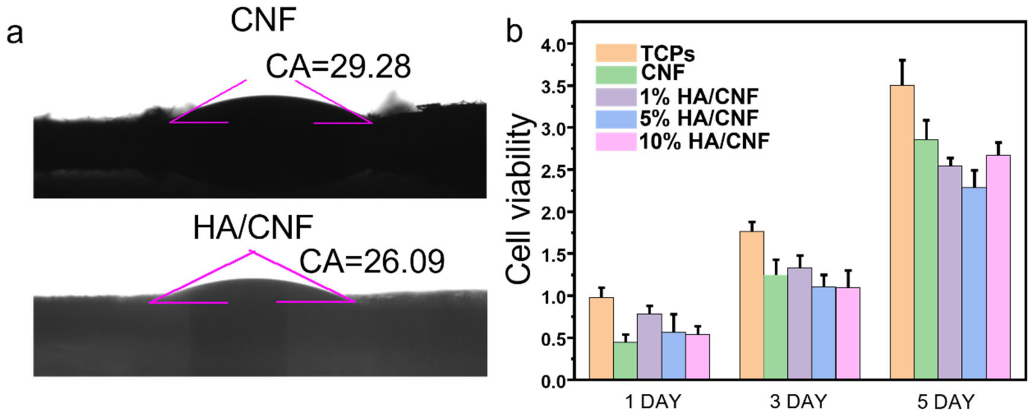

Figure S6. (**a**) Contact angle image of CNF and HAp/CNF. (**b**) Cell viability of different materials at 1, 3, and 5 days.

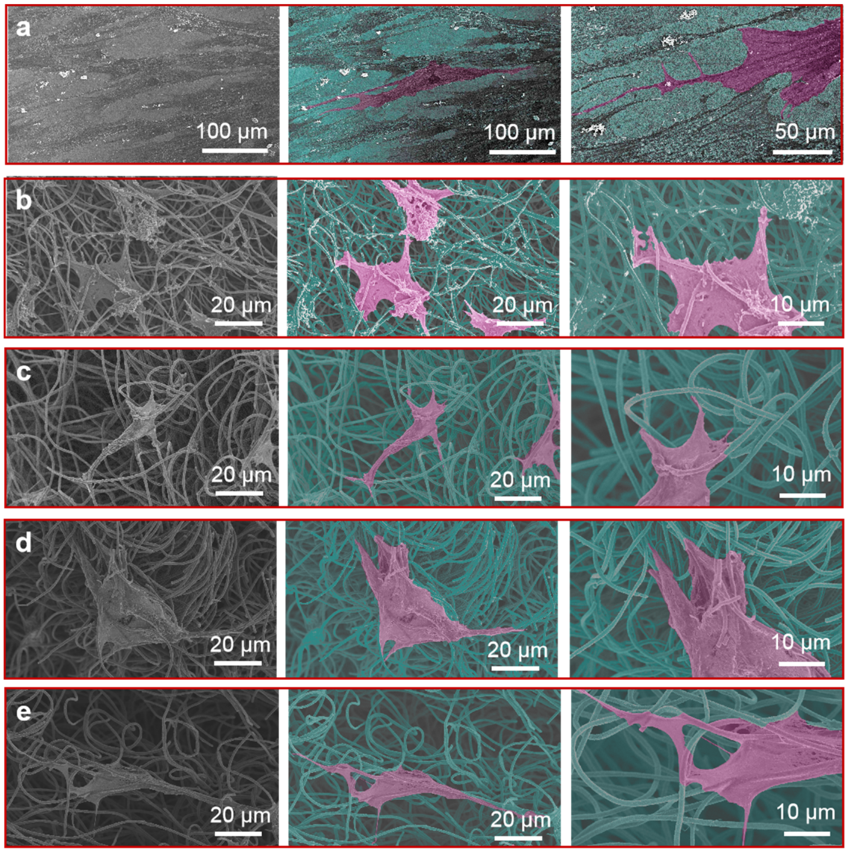

Figure S7. SEM images of h-ADSCs cultured for 7 days on CNF and HAp/CNF. (**a**) tissue culture plastic. (**b**) CNF membranes. (**c**) 1% HAp/CNF membranes. (**d**) 5% HAp/CNF membranes. (**e**) 10% HAp/CNF membranes.

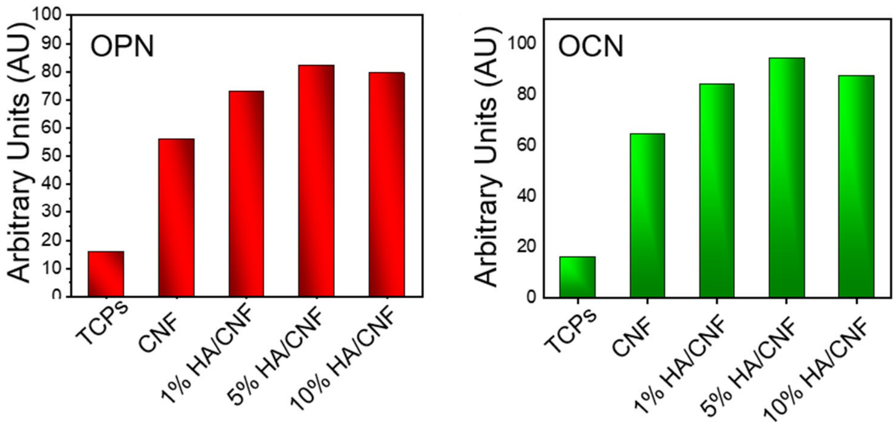

Figure S8. Statistics of cell immunofluorescence intensity on TCPs (tissue culture plastic), CNF, and HAp/CNF.
